# Editorial: The role of epigenetic modification in MDSC differentiation and function

**DOI:** 10.3389/fimmu.2023.1177138

**Published:** 2023-03-14

**Authors:** Xinyu Tian, Lele Zhu, Jie Tian, Shengjun Wang

**Affiliations:** ^1^ Department of Laboratory Medicine, Nanjing Drum Tower Hospital, Nanjing University Medical School, Nanjing, China; ^2^ Department of Immunology, The University of Texas MD Anderson Cancer Center, Houston, TX, United States; ^3^ Department of Immunology, Jiangsu Key Laboratory of Laboratory Medicine, School of Medicine, Jiangsu University, Zhenjiang, China; ^4^ Department of Laboratory Medicine, The Affiliated People’s Hospital, Jiangsu University, Zhenjiang, China

**Keywords:** epigenetic modification, MDSCs (myeloid-derived suppressor cells), regulatory mechanism, autoimmune, antitumor immune response

Myeloid-derived suppressor cells (MDSCs) represent a population of heterogeneous cells consisting of a pathologic state of activation of monocytes and relatively immature neutrophils. MDSCs consist of two main subsets: polymorphonuclear myeloid-derived suppressor cells (PMN-MDSCs) and monocytic myeloid-derived suppressor cells (M-MDSCs) (1). In mice, MDSCs are identified as CD11b^+^Gr1^+^, in which PMN-MDSCs are CD11b^+^Ly6G^+^Ly6C^low^ and M-MDSCs are CD11b^+^Ly6G^-^Ly6C^high^ (2). In humans, PMN-MDSCs are defined as CD11b^+^CD33^+^CD14^-^CD15^+^ cells sharing the same phenotype with mature neutrophils. Different from PMN-MDSCs, human M-MDSCs can be easily separated from monocytes based on the different expressions of human leukocyte antigen-DR (HLA-DR) (3).

In addition to phenotype, immunosuppression is the major characteristic of MDSCs, which makes them different from mature monocytes and neutrophils. MDSCs can directly suppress T-cell response by releasing high levels of suppressive molecules. MDSCs can also indirectly inhibit the immune response by inducing regulatory T cells (Tregs), promoting the differentiation of T helper 17 (Th17) cells, and inhibiting the activation of natural killer (NK) cells (4, 5). Moreover, it has been identified that exosomes from MDSCs are capable of mediating the immune response and development of target cells (6). Based on these mechanisms, MDSCs induce an immune suppressive environment and promote immune escape.

Accumulation of MDSCs takes place alongside the same differentiation pathways as for granulocytes and monocytes. Multiple molecules contribute to the accumulation of MDSCs, and these molecules can be further divided into two groups, which are responsible for MDSC expansion and activation, respectively (7). In the process of these molecules regulating MDSCs, epigenetic regulators play an important role. Epigenetic modifications represent the phenomenon of phenotype change in the absence of genotype change, which contains stable and transient structural changes of chromatin regions for registration, signaling, or perpetuating altered activity states. Chromatin changes play a central role in physiological and pathological procedures by modulating environmental signals and gene expression patterns. Epigenetic modification mainly consists of DNA modifications, histone modifications, chromatin remodeling, and non-coding RNAs (ncRNAs), which are heritable and reversible (8). Although it has been widely identified that epigenetic modifications induce changes in MDSC differentiation and function, the accurate regulatory mechanisms are still incompletely understood.

The goal of this Research Topic is to provide a forum to advance research on the accurate mechanisms that epigenetic modifications induce the changes in MDSC biology, as well as explore the clinical application prospect of epigenetic therapy targeting MDSCs in individuals with cancer or autoimmune disease. We focus on: (a) Mechanisms of epigenetic modifications that induce the changes in MDSC differentiation and function; (b) Clinical applications of epigenetic therapy targeting MDSCs; (c) Inducers of the epigenetic modification in MDSCs; (d) Cell biology changes of different MDSC subsets; (e) The influence of epigenetic modification in MDSCs on the intercellular interaction.

In this collection of articles, Xu et al. provide a detailed summary of the current research on the regulatory roles of DNA methylation, histone modifications, and non-coding RNAs in the development and immunosuppressive activity of MDSCs, and further summarize the distinct role of MDSCs in the pathogenesis of autoimmune diseases, to provide help for the diagnosis and treatment of diseases from the perspective of epigenetic regulation of MDSCs.

Xu et al. summarize the crosstalk between the epigenetic alterations and MDSC functions and briefly introduce how the accumulation and function of MDSCs caused by epigenetic modification impact the disease development, which represents a promising therapeutic strategy for the related disorders.

Zulqarnain et al. assess the effectiveness of endoscopic retrograde appendicitis therapy (ERAT) as a new technique and method for chronic fecalith appendicitis complicated by active ulcerative colitis. They find that ERAT can be seen as a different approach and be favored as a safer and more effective option in treating UC patients with appendicitis, especially those who are later in the course of the disease. Because of the ERAT procedure, such cases can avoid surgery and surgery-related complications.

Zhao et al. demonstrate the existing studies of MDSCs in IBD reporting either its pro-inflammatory roles or anti-inflammatory roles, along with the application of MDSCs targeted therapies in IBD. The rich diversity of topics discussed in this Research Topic is an indication of the many emerging roles of epigenetic modifications in MDSCs.

## Author contributions

The authors contributed equally and in mutual agreement to the reviews of the submitted manuscripts and the writing of the editorial. All authors contributed to the article and approved the submitted version.
